# A 5-year review of prevalence, temporal trends and characteristics of individuals experiencing moderate and severe food insecurity in 34 high income countries

**DOI:** 10.1186/s12889-023-17139-9

**Published:** 2023-11-09

**Authors:** Michelle L. Gatton, Danielle Gallegos

**Affiliations:** 1 Centre for Immunology and Infection Control, Faculty of Health, Queensland University of Technology (QUT), Victoria Park Rd, Kelvin Grove, QLD 4059 Australia; 2 School of Exercise and Nutrition Sciences, Faculty of Health, Queensland University of Technology (QUT), Victoria Park Rd, Kelvin Grove, QLD 4059 Australia; 3 Centre for Childhood Nutrition Research, Faculty of Health, Queensland University of Technology (QUT), Graham St, South Brisbane, QLD 4101 Australia

**Keywords:** Food insecurity, High income countries, Temporal trend, Prevalence, Severe food insecurity

## Abstract

**Background:**

Due to the relatively low numbers of households in high income countries experiencing food insecurity most studies conflate the levels of severity, which masks between- and within-country differences. This study aims to describe the characteristics of individuals living in high income countries who were moderately or severely food insecure and investigates temporal trends in prevalence. It assesses these characteristics in comparison to those who were food secure.

**Methods:**

This is a secondary analysis of data collected by the FAO Voices of the Hungry between 2014–2018. The data were collected during the annual Gallup World Polls of nationally representative samples using the Food Insecurity Experience Scale. Data from 34 highly developed, wealthy countries were analysed. The age, gender, income, education, area of residence and household structure of individuals experiencing moderate/severe food insecurity (FI), and severe FI, were compared using ANOVA, Welch’s F, Pearson’s Chi-square, and Linear-by-Linear Association, dependent on the variable of interest. Hierarchical cluster analysis was used to group countries according to their prevalence of moderate/severe FI, and severe FI.

**Results:**

Overall, 6.5% of the weighted sample were moderately/severely food insecure (M-SFI), while 1.6% were severely food insecure. M-SFI individuals were present in all 34 countries, in all years and across all education levels and income quintiles. The proportion of individuals experiencing moderate/severe FI varied between years and countries. Fifteen countries showed a significant downward temporal trend in prevalence of moderate/severe FI (*p* < 0.001), while three countries demonstrated an increasing temporal trend driven by increasing prevalence in those aged 65 years or less (*p* < 0.001). Comparing individuals experiencing moderate versus severe FI showed over-representation of males, single adult households and lower household income in the severe FI group.

**Conclusions:**

Individuals across all income, education and age categories living in high income countries are experiencing moderate/severe food insecurity, but with higher prevalence in those experiencing more disadvantage. Over the study period some countries experienced escalating while others demonstrated decreasing moderate/severe FI trends. This comparison of countries with similar economic and human development indices highlights an opportunity to investigate subtle variations in social, economic and education policy that could have profound impacts on food insecurity.

**Supplementary Information:**

The online version contains supplementary material available at 10.1186/s12889-023-17139-9.

## Background

The most recent internationally accepted definition of household food and nutrition security has been defined by the Food and Agriculture Organization (FAO) as existing when “all people at all times have physical, social and economic access to food, which is safe and consumed in sufficient quantity and quality to meet their dietary needs and food preferences … allowing for a healthy and active life” [1]. The definition is conceptualised as having a number of domains, including availability (nutritious affordable food is available for consumption); access (encompassing not only economic but also physical and social access); utilisation (which includes the equipment, resources and food literacy necessary to use food effectively) [2]. For high income, industrialised countries the availability of adequate quantities of food to meet caloric needs for a majority of the population is usually not an issue [3]. However, certain sectors of these populations still struggle to provide enough food of sufficient quality for an active and healthy life [4]. In most high income countries, the inability to put food on the table is largely due to neoliberal policies that result in low wages, insecure and unstable employment, minimal or inadequate social protection, unaffordable housing and unaffordable food [5]. Consequently, investigating the continuum of food security experiences is essential to evaluate both reduced quality and quantity of foods that individuals and households can access.

Most data on household food insecurity (HFI) in a high-income country (HIC) context has emerged from the United States of America, followed by Canada and the United Kingdom [6, 7]. More recently data is emerging from Australia, New Zealand, Europe and the Republic of Korea [7–10]. The USA and Canada are the only two countries that have regular population monitoring. The USA monitors HFI annually using the USDA Household Food Security Survey Module (HFSSM) [11] while Canada has biannual monitoring of some states using an adapted version of the same tool [12].The USDA HFSSM tool classifies food security based on severity which includes marginal (where there is anxiety/worry), low (when diet quality is compromised) and very low (when the quantity of food consumed is compromised [9].

Data from the USA and Canada report similar prevalence rates of HFI. In Canada in 2021, the prevalence of HFI was estimated to be 11.6% of which 4.2% were identified as severe (where household members had cut the size or skipped meals) [13]. The most recent prevalence estimate of HFI in the USA (2021) was 10.2% of which 3.8% had very low food security (equivalent to severe food insecurity in the Canadian context) [11]. In the UK, use of foodbanks is taken as a proxy indicator of food insecurity in the absence of regular monitoring; from 2015 to 2019 the Trussell Trust indicated a 73% increase in the use of food banks, although the proportion of the population using food banks at either date is unknown [14]. In Europe, again in the absence of regular monitoring, analysis of the Eurostat database indicated that 10.9% of participants in 2012 were not able to afford meat every second day, a proxy for uncertain or insufficient food availability and access arising from resource constraints [15].

In Australia, ad hoc national data relies on a single – two-part question which asks if in the last 12 months was there any time when they, or members of their household ran out of food, and could not afford to buy more. For those who answered affirmatively the second questions asks if they or members of their household had gone without food [16]. This question identifies the prevalence of food insecurity in 2011–12 to be around 4% [17]. This question has been demonstrated to underestimate HFI by between 5–8 percentage points [18]. In Japan there is no routine measurement of HFI and the national poverty line has been deployed as a proxy indicating 15.7% of the population are potentially food insecure [19]. In Korea the USDA HFFSM was deployed in 2012 and indicated a HFI prevalence of 11.3%, 2.0% with hunger [20]. In Denmark a representative sample of households using the 6 item USDA HFFSM revealed a prevalence of low and very low food security as 6.0% and 2.4% respectively [21]. Other countries have used different ad hoc measures, or no measures at all (see for example New Zealand) [22]. The disparity in measurement makes it difficult to undertake cross-country comparisons. This recognition across all countries led to the development of the FAO Food Insecurity Experience Scale (FIES) (Voices of the Hungry [VoH]) that enables comparison at an individual level across different levels of severity.

Households and individuals with low economic resources and poor labour market attachment appear to be most at risk of food insecurity (FI) in HIC [23]. Analysis of the FIES data across 147 countries identified that although demographics factor into determining FI in HIC, social and economic variables are potentially more relevant [24]. Other analyses of the same data indicated that FI in HIC was associated with age and number of child in the household, being single, separated, having an elementary and secondary education, residing in a rural area, log household income, and employment status ( part-time employment, unemployment) [25]. It appears that those at risk of HFI in more industrialised nations are: households with children led by single parents [25], single person households [24, 25], low income households (but increasingly middle income as well) [23, 24, 26], those receiving welfare payments [23], Indigenous (Aboriginal and Torres Strait Islander in Australia, Māori in New Zealand, First Nations populations in the USA and Canada) [27–29], refugees and migrants [30, 31], and people living with a disability or chronic conditions [32].

Other studies using the FIES data either in HIC or longitudinally include analyses of countries located in the Middle East [33], in Latin America [34] and in central and Eastern Europe. Omidvar et al. [33] investigated rich and politically stable countries in the Middle East (Lebanon, Emirates, Kuwait, Saudi, Bahrain) finding that the odds of being food insecure were significantly elevated among individuals aged 13 to 25 years (OR = 1.858), living in the poorest quintile (OR = 4.317), and with low education attainment (OR = 1.354). Personal health (OR = 2.958), social capital (OR = 1.899), and not enough money for shelter (OR = 4.859) were all found significantly associated with FI. Providing insights into changes in FI status from 2014–2017, De Sousa et al. [34] investigated rates of FI in Latin American countries classifying them as improving, worsening or stagnating. Finally, in central and eastern Europe (Lithuania, Slovakia and Poland) the FIES data were used to identify between country differences in prevalence and determinants [35].

Due to the relatively low numbers of households in HIC experiencing FI most studies conflate the levels of severity, typically dichotomising the sample to food secure and food insecure. The FAO data is the first opportunity to explore a pooled sample of households who are experiencing severe FI in HIC, that is where individuals are reducing the quantity of food they consume, due to a lack of financial or other resources. With similar developed social welfare, employment, agricultural, food retail, transport systems and policies, such an exploration will allow elucidation of between and within-country factors that may be impacting on the manifestation of severe FI. To our knowledge there are no published studies investigating, in depth, FI across the levels of severity in HIC, over time. Thus, the current analysis aims to describe the characteristics of individuals who were moderately or severely food insecure and to assess temporal trends in the prevalence of moderate and severe FI between 2014 and 2018.

## Methods

This study is a secondary data analysis of data obtained under licence from FAO-VoH.

### Sample

Data were collected during the 2014–2018 Gallup World Polls (GWP). Each GWP is a nationally representative sample of approximately 1000 individuals aged ≥ 15 years. The GWP is typically conducted annually in over 140 countries, maintaining a core set of questions across surveys. Detailed sampling and data collection methodology, along with data preparation has been previously reported [36]. Data weighting has been applied, with weights adjusting for oversamples and household size, as well as post-stratification weighting using population statistics for gender, age, and potentially education or socioeconomic status where reliable data were available [36].

### Geographic scope

This study only considered data from highly developed, wealthy countries. The categorisation has been made based on a combination of the World Banks’s index of Gross National Income (GNI) and the United Nations Human Development Index (HDI). Only countries with a GNI above USD12,500 [37] and a HDI above 0.8 are included [38]. Thirty-four countries from seven regions were included; Oceania (*n* = 2), Western Europe (*n* = 10), Central Europe (*n* = 7), Eastern Europe (*n* = 6), Nordic region (*n* = 4), East and South-East Asia (*n* = 3), and North America (*n* = 2).

### Food insecurity measures

Food insecurity measures were derived by the FAO-VoH based on responses to the eight questions in the FIES. The FIES questions, scales and how severity is calculated are all available at the FAO Voices of the Hungry website (https://www.fao.org/in-action/voices-of-the-hungry/en/). To allow for multi-country analysis, moderate/severe FI, and severe FI, was defined using the probability of moderate or severe FI and the probability of severe FI, respectively. These probability measures take into account measurement error and are based on adjustment of respondent severity parameters to the VoH global standard, so are intended to be comparable between countries [39]. The threshold of severe FI is specified at the severity level of the FIES item “did not eat for a whole day”, while moderate/severe FI is specified at the level of severity associated with the FIES item “ate less than should”. In both cases, individuals with a probability of moderate/severe FI, or severe FI, ≥ 0.50 were classified as being moderately or severely, or severely food insecure, respectively.

### Social and demographic variables

Selected social and demographic variables collected during the GWP were included in this analysis including participant age, gender, income quintile (within the country), per capita annual income in International Dollars (Int’l$), education level, number of adults living in household, number of children living in household and area of residence (urban/suburbs vs towns/rural). A new variable was created called ‘household structure’ based on the number of adults and children living in the household: single adult with no children, single adult with children, two adults with no children, two adults with children, three or more adults with no children, and three or more adults with children.

### Statistical analysis

The characteristics of individuals experiencing moderate/severe FI, and severe FI, were compared using ANOVA, Welch’s F, Pearson’s Chi-square, and Linear-by-Linear Association, dependent on the variable of interest. Where a significant association was indicated using Pearson’s Chi-square, the z-test with Bonferroni correction was used to compare proportions. The mean age was compared across income quintiles using Welch’s F, with Dunnett’s T3 used for post-hoc tests due to unequal variances between the groups.

Hierarchical cluster analysis was used to group countries according to their prevalence of moderate/severe FI, and severe FI. Exploratory analysis of the temporal trends in the proportion of individuals experiencing moderate/severe FI, and the proportion experiencing severe FI, using generalized linear mixed models with a Poisson link function indicated that country was an effect modifier of the temporal trend for both moderate/severe FI and severe FI. As a result, country-by-country analyses were conducted using the Linear-by-Linear statistic to assess linear trends, and Pearson’s Chi-square to detect any differences between individual years within each country.

Association between the prevalence of moderate/severe FI and severe FI within a country was tested using Pearson’s Correlation Coefficient. All statistical analysis was conducted using SPSS Version 27 (IBM Corp).

## Results

A total of 154,704 individuals from 34 countries had a food insecurity classification and were included in the analysis. Data for all 34 countries were available for 2014–2017, while 22 countries had data for 2014–2018. The countries missing 2018 data were Australia, Belgium, Cyprus, Finland, Germany, Italy, Luxembourg, New Zealand, Norway, South Korea, Sweden and United States. For some variables, 2018 data from the United Kingdom was also missing.

**Fig. 1:**
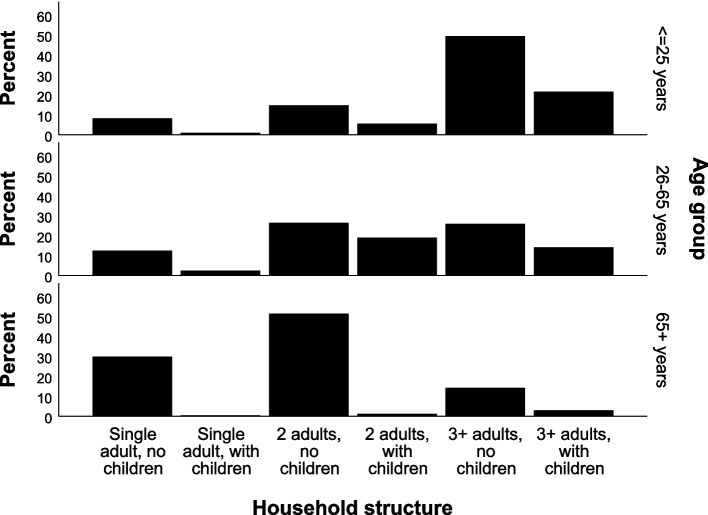
Profile of household structure within each age group

Overall, the weighted sample was 51.6% female, with mean age 46.6 years (sd 18.4; interquartile range (IQR) 32.0 – 61.0 years) and median per capita annual income of Int’l$ 12,266 (IQR 7,155 – 21,204). Approximately 16% (15.7%) were aged 25 years or less, while 18.3% were aged over 65 years. Over 70% (71.4%) of the weighted sample lived in adult-only households, with 14.9% living alone. Approximately 2% (1.8%) lived in single adult households with children. The household structure varied across age groups with 29.9% of those aged 65 + years living alone, compared to 8.1% and 12.4% for ≤ 25 years and 26–65, respectively (Fig. 1). Almost 71% of those aged ≤ 25 years lived in households with 3 + adults, either with (21.5%) or without (49.3%) children.

### Moderate or severe food insecurity

#### Prevalence

**Fig. 2:**
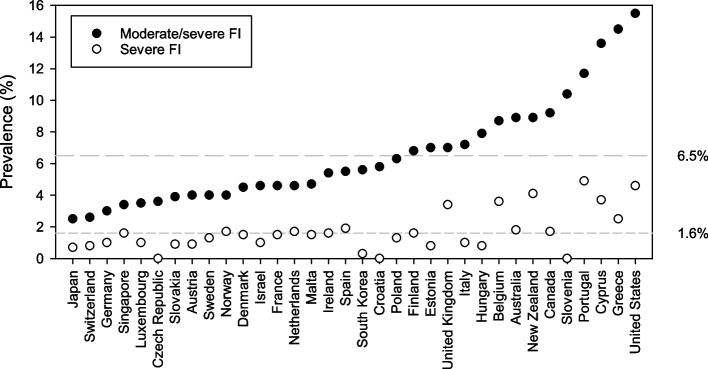
Prevalence of moderate and severe food insecurity by country, 2014–2018

Overall, 6.5% of the weighted sample were moderately/severely food insecure (M-SFI). M-SFI individuals were present in all 34 countries, in all years and across all education levels and income quintiles. The prevalence of moderate/severe FI ranged from 2.5% in Japan to 14.5% in Greece and 15.5% in the United States (Fig. 2, Supplemental Table 1 in Additional file 1). Hierarchical cluster analysis suggested that countries could be assigned to one of four groups based on the prevalence rate; Group 1 – United States, Greece and Cyprus (> 13%), Group 2 – Portugal, Slovenia, Hungary, Canada, Belgium, New Zealand and Australia (8–12%), Group 3 – Spain, South Korea, Ireland, Croatia, Estonia, United Kingdom, Italy, Finland and Poland (5–7%), and Group 4 – Czech Republic, Luxembourg, Slovakia, Austria, Norway, Sweden, Malta, Denmark, Israel, Netherlands, France, Singapore, Germany, Switzerland and Japan (< 5.0%).

#### Characteristics of individuals who were moderately or severely food insecure

Fifty-six percent of individuals experiencing moderate/severe FI were female, 52% were unemployed or out of the workforce and 47% were in the poorest income quintile (Table 1). One in five (21%) M-SFI individuals lived alone, while 35% had children in the household (Table 1). For individuals aged 25 years or less, a significantly higher proportion of M-SFI individuals lived in single or two-adult households, with or without children, compared to individuals who were not M-SFI (*p* < 0.001). This was offset by a lower proportion living in 3 + adult households with no children (30.7% for M-SFI compared to 50.6% for not M-SFI). For those aged 26–65 years, single adult households (with or without children), and households with 3 + adults with children were over-represented amongst the M-SFI group, compared to those who were not M-SFI (*p* < 0.001; 42% compared to 28%). For those over 65 years, single adult households with no children were significantly over-represented in the M-SFI group (39.2% vs 29.5%), while those in two-adult households (no children) were under-represented (38.9% vs 51.9%) (*p* < 0.001).

**Table 1:** Characteristics of individuals according to their food insecurity status ^1,2^

	**Not moderate/severe FI (*n* = 144,571)**	**Moderate/severe FI (*n* = 10,058)**	**Severe FI**^**a**^ **(*n* = 2,525)**
Mean probability of moderate/severe FI (sd; IQR)	0.02 (0.07; 0.00 – 0.00)	0.90 (0.12; 0.76 – 0.99)	0.99 (0.004; 0.99 – 1.00)
Mean probability of severe FI (sd; IQR)	0.00 (0.00; 0.00 – 0.00)	0.25 (0.30; 0.00 – 0.50)	0.70 (0.12; 0.60 – 0.80)
**Demographics**			
Mean age (sd; IQR)	46.7 (18.5; 32.0 – 61.0)	44.2 (16.7; 31.0 – 56.0)* ^**c**^	43.2 (15.7; 31.0 – 54.0)
Aged ≤ 25 years (%)	15.7	15.6	15.2
Aged over 65 years (%)	18.8	11.9* ^**d**^	9.3
Female (%)	51.3	55.7* ^**d**^	51.6
% living in Urban/suburbs	46.0	47.9* ^**d**^	48.9
**Employment status**^**b**^			
Full-time (%)	47.3	34.7* ^**d**^	31.0
Part-time (do not want full-time) (%)	7.7	4.6* ^**d**^	3.9
Part-time (want full-time) (%)	5.0	8.7* ^**d**^	8.7
Unemployed (%)	3.8	14.5* ^**d**^	17.6
Out of workforce (%)	36.2	37.6* ^**d**^	38.7
**Marital status**^**b**^			
Single/never married (%)	26.7	32.8* ^**d**^	37.5
Married/domestic partner (%)	60.6	45.9* ^**d**^	39.6
Separated/divorced (%)	6.4	14.1* ^**d**^	17.1
Widowed (%)	6.3	7.1* ^**d**^	5.8
**Household structure**			
Mean number adults in household (sd; IQR)	2.5 (1.2; 2.0 – 3.0)	2.4 (1.3; 1.0 – 3.0)* ^**c**^	2.3 (1.4; 1.0 – 3.0)
Mean number children in household (sd; IQR)	0.5 (0.9; 0.0 – 1.0)	0.7 (1.1; 0.0 – 1.0)* ^**c**^	0.7 (1.2; 0.0 – 1.0)
Households with ≥ 3 children (%)	3.4	7.4* ^**d**^	8.7
**Education**			
≤ 8 years (%)	16.6	27.6* ^**d**^	29.0
9–15 years (%)	61.1	62.8* ^**d**^	62.4
College degree (%)	22.3	9.6* ^**d**^	8.6
**Income**			
Median annual household income in Int’l$ (IQR)^b^	18,000 (7,800 – 57,000)	16,160 (9,998 – 27,140)* ^**e**^	14,415 (9,045 – 23,728)
**Income quintile**			
Poorest (%)	17.7	46.9* ^**d**^	54.2
2^nd^ poorest (%)	19.7	23.7* ^**d**^	22.3
Middle (%)	20.3	14.6* ^**d**^	11.6
2^nd^ richest (%)	20.9	9.9* ^**d**^	8.4
Richest (%)	21.3	4.9* ^**d**^	3.4
**Household structure**			
Single adult, no children (%)	14.5	21.3* ^**d**^	25.6
Single adult, with children (%)	1.6	4.4* ^**d**^	6.0
2 adults, no children (%)	29.6	22.2* ^**d**^	20.4
2 adults, with children (%)	13.5	14.2	13.8
3+ adults, no children (%)	27.8	21.5* ^**d**^	16.8
3+ adults, with children (%)	13.0	16.4* ^**d**^	17.4

^1^Weighted sample sizes for individual variables vary by up to 2% due to missing data

^2^The moderate/severe FI group was compared to those who were not moderate/severe FI

^a^Severe FI individuals are a subset of individuals within the moderate/severe FI group

^b^Excludes data from UK 2018

^c^Welch’s unequal variances t−test

^d^Chi−square test of Independence, with z test (Bonferroni corrected) to compare proportions between categories

^e^Mann−Whitney U t−test

* *p*<0.001

#### Country-specific age prevalence rates

**Fig. 3:**
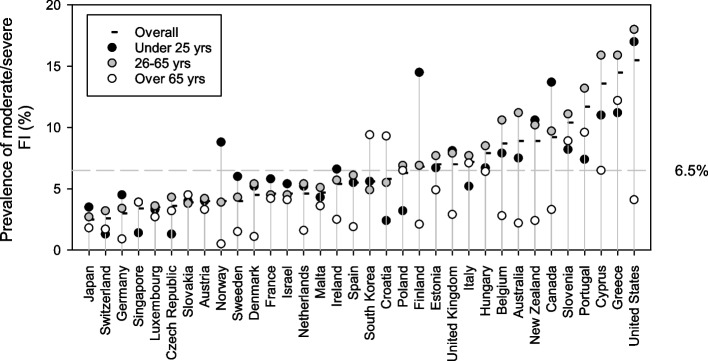
Country age-specific prevalence of moderate/severe FI, 2014–2018

Across the 34 countries the prevalence rate for moderate/severe FI was 6.5%, 7.2% and 4.2% for individuals aged 25 years or less, 26–65 years and 65 + years, respectively. Age prevalence rates and patterns varied between countries (Fig. 3, Supplemental Table 1 in Additional file 1). The United States had the highest prevalence of moderate/severe FI among individuals aged 25 years or less (17.0%), and 26–65 years (18.0%), while Greece reported the highest prevalence among those aged 65+ years (12.2%). The oldest age group typically had the lowest prevalence of moderate/severe FI, except in Croatia and South Korea where the prevalence in this age group was almost double that for individuals aged 26–65 years (Supplemental Table 1 in Additional file 1). The prevalence for those aged 25 years or less was generally lower or similar to the prevalence for the 26–65 year age group, but there were three clear exceptions: Canada (13.7% vs 9.7%), Finland (14.5% versus 6.9%) and Norway (8.8% versus 3.9%) (Fig. 3, Supplemental Table 1 in Additional file 1).

#### Temporal trends

**Fig. 4:**
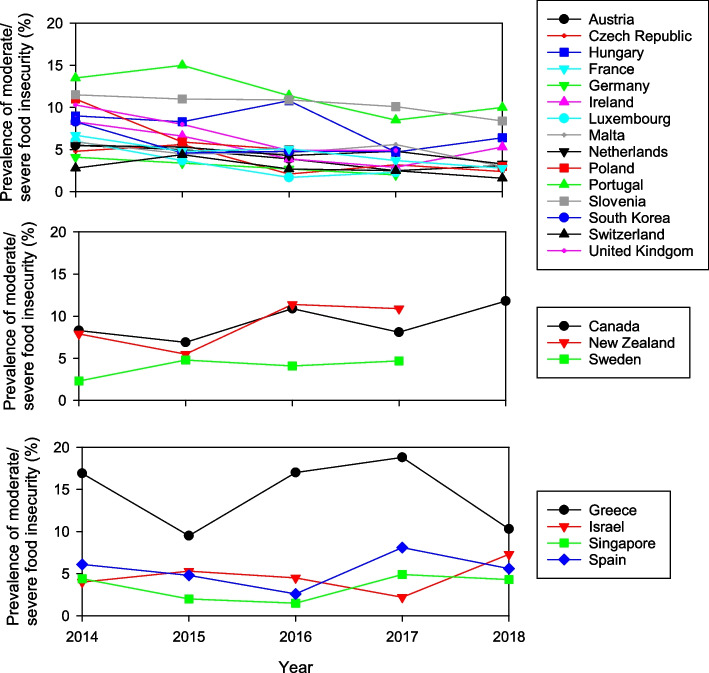
Temporal trends in proportion of individuals experiencing moderate/severe FI for countries with significant linear decrease (top), linear increase (middle) and no linear trend (bottom)

The proportion of individuals experiencing moderate/severe FI varied between years, from 7.4% (2014) to 5.4% (2018). Overall, there was a significant linear trend for declining prevalence of moderate/severe FI (*p* < 0.001), but the temporal trends differed between countries (*p* < 0.001). Twelve of the 34 countries demonstrated no significant trend or difference in the prevalence of moderate/severe FI between years (*p* > 0.05), while 15 countries showed a significant linear trend for decreasing prevalence (*p* < 0.03). Of the remaining countries, three (Sweden, Canada and New Zealand) showed a significant trend for increasing prevalence, and four had significant differences detected between years without a consistent trend (Fig. 4).

**Fig. 5:**
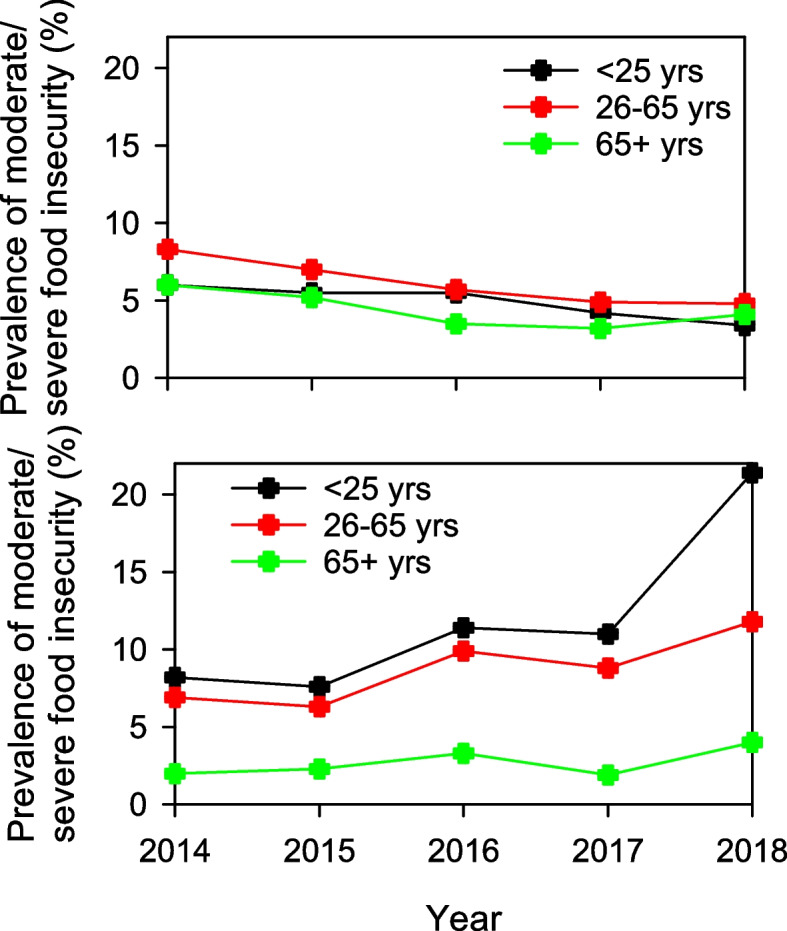
Temporal trends in proportion of individuals experiencing moderate/severe FI, by age group between 2014 and 2018 for countries with an overall decreasing trend (*n* = 15) (top) and countries with an overall increasing trend (*n* = 3) (bottom)

For the 15 countries with an overall decreasing trend, this trend was significant within each age group (*p* < 0.001). In contrast, the increasing trend identified for Canada, New Zealand and Sweden was restricted to those aged 25 years or less (*p* < 0.001) and 26–65 years (*p* < 0.001), with no trend for those aged 65 + years (*p* = 0.359). This result did not change when 2018 data was excluded from the analysis (due to 2018 data only being available for Canada). Notably, the prevalence of moderate/severe FI in countries with a decreasing trend was similar between the three age groups, whereas countries with an increasing trend showed higher prevalence of moderate/severe FI in those aged 65 years or less (Fig. 5).

### Severe food insecurity

#### Prevalence

Almost 2% (1.6%) of individuals were classified as severely food insecure. These individuals were distributed across 31 of the 34 countries, with no severely food insecure individuals identified in Croatia, Czech Republic or Slovenia. There was a strong significant linear correlation between the prevalence of moderate/severe FI and the prevalence of severe FI within a country (*r* = 0.65, *p* < 0.001).

Countries could be grouped into four categories based on the prevalence of severe FI, using hierarchical cluster analysis. These groups were: Group 1 – Portugal, United Kingdom, Belgium, United States, New Zealand and Cyprus (> 3.4%), Group 2 – Greece (2.5%), Group 3 – Malta, Poland, Ireland, Netherlands, Singapore, Italy, Japan, Austria, Luxembourg, Hungary, Slovakia, Switzerland, Germany, Sweden, Estonia, Israel, Finland, France, Norway, Denmark, Australia, Spain and Canada (0.7—1.9%), and Group 4 – Czech Republic, Slovenia, Croatia and South Korea (< 0.4%).

#### Characteristics of individuals who were severely food insecure

Approximately half of the severely food insecure individuals were female, lived in urban/suburban areas and were in the lowest income quintile for their country (Table 1). Approximately 55% of severely food insecure individuals were single, divorced/separated or widowed.

#### Country-specific age prevalence rates

The highest prevalence of severe FI was 4.9% in Portugal (Fig. 2). Age-specific prevalence rates for severe FI exceeded 5% for individuals aged 25 years or less in New Zealand (6.0%) and United States (5.6%), and for individuals aged 26–65 years in Portugal (5.5%) and United States (5.4%) (Supplemental Table 1 in Additional file 1).

#### Temporal trends

**Fig. 6:**
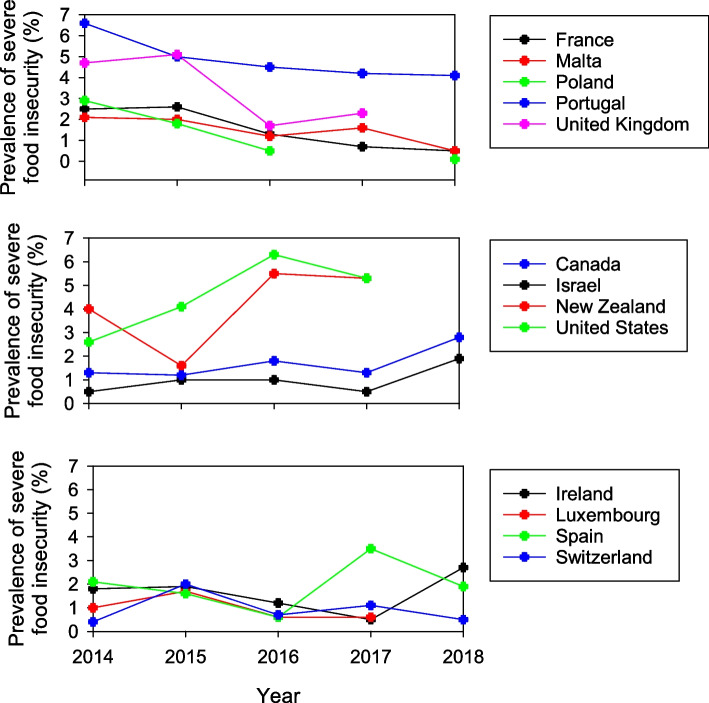
Temporal trends in proportion of individuals experiencing severe FI, between 2014 and 2018 for countries with significant linear decrease (top), linear increase (middle) and no linear trend (bottom)

Overall, there was a slight but significant decreasing linear trend in the prevalence of severe FI over the study period (*p* = 0.014). Prevalence peaked at 1.8% in 2015, declining to 1.3% in 2018. Analysis of the temporal trend in the prevalence of severe FI revealed no change over time for 18 countries, a decreasing trend for five countries, an increasing trend for four countries, and sporadic differences between years in the remaining four countries (Fig. 6).

### Comparison of moderately and severely FI individuals

One quarter (25.1%) of the M-SFI individuals were classified as severely FI. This value differed between countries, ranging from 0% in Croatia, Czech Republic and Slovenia, to 46% in Singapore and New Zealand, and 47% in United Kingdom. A higher proportion of males were severely FI, compared to females (27.4% versus 23.3%, *p* < 0.001), with severely food insecure individuals slightly younger than moderately food insecure individuals (mean difference 1.3 years, 95% CI: 0.6—2.0 years, *p* < 0.001). Over 30% (31.5%) of severely food insecure individuals lived in single adult households (with and without children), compared to 23.8% for moderately food insecure individuals (*p* < 0.05), while they were under-represented in multiple adult households without children (37.2% for severely food insecure compared to 45.8% for moderately food insecure, *p* < 0.05). The annual household income for severely food insecure individuals (Int’l$ 20,532, 95% CI: Int’l$ 19,406 – 21,656) was significantly lower than for moderately food insecure individuals (Int’l$ 26,131, 95% CI Int’l$ 24,157 – 28,105) (*p* < 0.001).

## Discussion

This analysis of the FAO-VoH data has shown that 6.5% of individuals are estimated to be moderate or severely food insecure, and 1.6% severely food insecure in HIC globally. Moderate/severe FI occurred across all ages, income quintiles and education levels, however those living in single adult households and those with lower household incomes were more likely to be severely food insecure. Preliminary analysis comparing moderate with severe FI indicated no statistically significant difference in per capita income. This indicates that income needs to be contextualised to the individual household’s living arrangements, and that FI severity in a HIC context is highly sensitive to income. In Europe, for example, each 1% rise in unemployment was associated with a 0.29 percentage point rise in the prevalence of FI, and each $1000 decrease in annual average wages was association with a 0.62 percentage point increase in FI [40]. The country with the highest prevalence of severe FI in the current analysis (4.9%) was Portugal, where from 2011 social support and benefits were reduced as unemployment and poverty increased [41].

Given that income is critical for minimising FI, population level measures to protect and ensure stable incomes including ensuring social protection and wages that support a decent standard of living are needed. Ensuring a living wage, irrespective of social circumstances, has the potential to ameliorate severe food insecurity, that is going without food. In this study, three countries (Czechia, Slovenia and Croatia) had no severely FI individuals and it is interesting to note that all operate under a post-socialist welfare model, in the Bismarckian tradition [42]. In addition, Slovenia made changes to minimum wage legislation starting in 2007 in response to the increasing price of food, electricity and other essential consumer goods, leading to legislative changes in 2018 resulting in a true living wage [43]. This corroborates the findings from Reeves et al. (2017) that indicated the probability of severe FI was dependent on how wages were established with little or no wage setting policy associated with higher odds of severe FI [44]. For those who were unemployed, the prevalence of severe FI did not change across the models indicating that reducing unemployment and ensuring a dignified social protection system all need to be considered [44].

For 44% of the HIC countries included in this analysis there was evidence of improvements in the prevalence of moderate/severe FI over time. Countries that experienced a linear increase in moderate/severe FI from 2014 to 2018 included Canada, New Zealand and Sweden, while the USA and Israel also had an increasing trend in the prevalence of severe FI. It is unclear why this was the case, but the observation of differences in prevalence of moderate/severe FI between age groups in these countries contrasts the more uniform prevalence seen between age groups in countries with a decreasing trend. In Canada, NZ and Sweden, the burden of moderate/severe FI appears to fall on the younger age groups, and it is an increasing prevalence in those younger than 65 years that is causing the overall increasing trend. Differences in trends between countries, and age groups, suggest complex intersections between social, income and food policies, all of which are subject to varying levels of political upheaval and change.

When looking at the socio-demographics of individuals more likely to report moderate/severe FI in HIC these were: female, those living in single adult households (with or without children) and in the lowest income quintile. This agrees with other previously published studies [24, 25]. However, within those reporting moderate/severe FI, males were more likely than females to experience severe FI. This result highlights the value of analysing moderate and severe FI separately, using samples that are weighted to better reflect population characteristics such as those in the FAO-VoH data. A cross-sectional study in Australia (*N* = 1010 of which 46% of respondents were men) indicated a higher proportion of men experienced severe FI, were less likely to be FI as they got older but were more likely to be FI if they were single or lived alone with children [45]. The drivers of these gender differences could relate to any number of factors such as social networks, mental health, gendered support services and income, and warrant further investigation.

Patterns in prevalence rates across age groups differed in some countries which may be an indicator of different social supports, particularly in the oldest and youngest age groups. Of note, are the higher rates of severe food insecurity in those aged over 65 years in Greece, Croatia and South Korea. In other countries the old age pension appears to protect most seniors from financial food insecurity (see for example Canada and the USA) [46, 47]. When looking at younger people and FI, country variations on transitioning from secondary schooling to work or to further education and the associated costs and government support are likely to have an impact. Similarly, the proximity of further education facilities and the social norms associated with living away from the family home could impact those under 25 years of age. A recent review of FI among post-secondary school students in the USA, Canada, Australia and Poland estimated prevalence rates between 9–89% [26], much higher than the values in this study.

There is significant variability in how social protection and income policies are enacted across countries and this potentially contributes to food security status. In Australia those receiving welfare payments including JobSeeker (previously NewStart), Austudy/Abstudy (support for attending post-secondary education), Disability Support Pension, the Carer Payment and the Parenting payment were all more likely to be FI [23]. Reeves et al. [48] used the FAO-VoH, FIES data across 142 countries to compare the family policy impact on FI. They found that those countries that implement family policies (e.g. family benefits, child care subsidies) have lower FI prevalence, this was more prominent for families with children at the lower end of income distributions. Child-specific cash transfers were found to be more effective in reducing severe food insecurity [48].

This analysis has several strengths and limitations. It is, to our knowledge, the first to look at temporal and between country changes to FI in HIC using a valid, scalar equivalent measure. The FIES can estimate and monitor food insecurity experiences across countries irrespective of cultural and social differences [49]. The data has some limitations in that variations between countries could be an artefact of cultural norms and perceptions associated with stigma and shame. There is no measurement of HFI among those who may be at more risk for example, First Nations peoples, those who are homeless or experiencing deep deprivation. It should also be acknowledged that the analysis conducted used data pre-dating the COVID-19 pandemic, and that some of the trends reported may have subsequently been impacted by the sudden and significant social and economic impacts caused by the pandemic. Future research into the prevalence and trends during, and after the COVID-19 pandemic, would provide valuable information about the stability of food insecurity in HIC.

## Conclusion

The analysis of this data indicates that HIC are not immune from experiencing moderate and severe food insecurity at the individual level. Individuals across all income, education and age categories are affected but with prevalence of moderate/severe FI higher in those experiencing more disadvantage. There were no statistically significant differences in per capita income between moderate and severely food insecure individuals meaning that income needs to be contextualised to the individual household’s living arrangements. During the study period, some countries experienced escalating while others demonstrated decreasing moderate/severe FI trends. The complex socio-political drivers of these variable trajectories need further investigation. FI severity in a HIC context is highly sensitive to income. This comparison of countries with similar economic and human development indices highlights an opportunity to investigate subtle variations in social, economic and education policy that could have profound impacts on food insecurity.

### Supplementary Information

**Additional file 1: Supplemental Table 1.** Estimated prevalence of moderate/severe food insecurity (FI), and severe FI, in 34 countries, 2014-2018.

## Data Availability

This study is a secondary data analysis of data obtained under licence from FAO-VoH; part of the data is not publicly available, however the specific data used in this analysis may be provided upon request.

## Abbreviations

FAO: Food and Agriculture Organization
FI: Food insecurity
FIES: Food insecurity experience scale
GNI: Gross National Income
GWP: Gallup World Polls
HDI: Human development index
HFI: Household food insecurity
HFSSM: Household Food Security Survey Module
HIC: High income countries
M-SFI: Moderately/severely food insecure
VoH: Voices of the Hungry
