# Supplementary material for: A 5-year review of prevalence, temporal trends and characteristics of individuals experiencing moderate and severe food insecurity in 34 high income countries
Source: BMC Public Health. 2023 Nov 9;23:2215. doi: 10.1186/s12889-023-17139-9 (PMC10636923; doi:10.1186/s12889-023-17139-9)
Supplement: Supplementary file 1 — Additional file 1: Supplemental Table 1. Estimated prevalence of moderate/severe food insecurity (FI), and severe FI, in 34 countries, 2014-2018. [file 12889_2023_17139_MOESM1_ESM.docx]

Additional File 1

Supplemental Table 1. Estimated prevalence of moderate/severe food insecurity (FI), and severe FI, in 34 countries, 2014-2018.

| Country | Prevalence moderate/ severe FI (%) | Prevalence severe FI (%) | Age-specific prevalence of moderate/severe FI (%) | | | Age-specific prevalence of severe FI (%) | | |
| --- | --- | --- | --- | --- | --- | --- | --- | --- |
|  |  |  | ≤25 yrs | 26-65 yrs | 65+ yrs | ≤25 yrs | 26-65 yrs | 65+ yrs |
| Australia | 8.9 | 1.8 | 7.5 | 11.2 | 2.2 | 0.5 | 2.6 | 0.3 |
| Austria | 4.0 | 0.9 | 4.0 | 4.2 | 3.3 | 1.3 | 0.9 | 0.7 |
| Belgium | 8.7 | 3.6 | 7.9 | 10.6 | 2.8 | 3.5 | 4.4 | 1.2 |
| Canada | 9.2 | 1.7 | 13.7 | 9.7 | 3.3 | 1.6 | 1.9 | 0.7 |
| Croatia | 5.8 | 0.0 | 2.4 | 5.5 | 9.3 | 0.0 | 0.0 | 0.0 |
| Cyprus | 13.6 | 3.7 | 11.0 | 15.9 | 6.5 | 1.4 | 4.9 | 1.5 |
| Czech Republic | 3.6 | 0.0 | 1.3 | 4.3 | 3.2 | 0.0 | 0.0 | 0.0 |
| Denmark | 4.5 | 1.5 | 5.2 | 5.4 | 1.1 | 1.4 | 2.0 | 0.2 |
| Estonia | 7.0 | 0.8 | 6.7 | 7.7 | 4.9 | 0.7 | 0.9 | 0.4 |
| Finland | 6.8 | 1.6 | 14.5 | 6.9 | 2.1 | 3.8 | 1.6 | 0.4 |
| France | 4.6 | 1.5 | 5.8 | 4.5 | 4.2 | 2.1 | 1.2 | 2.0 |
| Germany | 3.0 | 1.0 | 4.5 | 3.4 | 0.9 | 1.7 | 1.1 | 0.2 |
| Greece | 14.5 | 2.5 | 11.2 | 15.9 | 12.2 | 2.0 | 2.8 | 1.8 |
| Hungary | 7.9 | 0.8 | 6.7 | 8.5 | 6.4 | 0.3 | 1.0 | 0.4 |
| Ireland | 5.4 | 1.6 | 6.6 | 5.7 | 2.5 | 1.5 | 1.8 | 0.9 |
| Israel | 4.6 | 1.0 | 5.4 | 4.5 | 4.1 | 1.0 | 1.0 | 1.0 |
| Italy | 7.2 | 1.0 | 5.2 | 7.7 | 7.1 | 0.8 | 0.9 | 1.4 |
| Japan | 2.5 | 0.7 | 3.5 | 2.7 | 1.8 | 1.3 | 0.7 | 0.5 |
| Luxembourg | 3.5 | 1.0 | 3.3 | 3.6 | 2.7 | 0.5 | 1.1 | 1.1 |
| Malta | 4.7 | 1.5 | 4.3 | 5.1 | 3.6 | 1.4 | 1.6 | 1.1 |
| Netherlands | 4.6 | 1.7 | 5.2 | 5.4 | 1.6 | 1.9 | 2.1 | 0.5 |
| New Zealand | 8.9 | 4.1 | 10.6 | 10.2 | 2.4 | 6.0 | 4.5 | 0.9 |
| Norway | 4.0 | 1.7 | 8.8 | 3.9 | 0.5 | 3.0 | 1.9 | 0.0 |
| Poland | 6.3 | 1.3 | 3.2 | 6.9 | 6.5 | 1.0 | 1.6 | 0.8 |
| Portugal | 11.7 | 4.9 | 7.4 | 13.2 | 9.6 | 2.3 | 5.5 | 4.5 |
| Singapore | 3.4 | 1.6 | 1.4 | 3.9 | 3.9 | 0.4 | 1.8 | 2.1 |
| Slovakia | 3.9 | 0.9 | 4.0 | 3.8 | 4.5 | 0.6 | 1.0 | 0.5 |
| Slovenia | 10.4 | 0.0 | 8.2 | 11.1 | 8.9 | 0.0 | 0.0 | 0.0 |
| South Korea | 5.6 | 0.3 | 5.6 | 4.9 | 9.4 | 0.4 | 0.3 | 0.4 |
| Spain | 5.5 | 2.0 | 5.5 | 6.1 | 1.9 | 1.2 | 2.4 | 0.3 |
| Sweeden | 4.0 | 1.3 | 6.0 | 4.3 | 1.5 | 2.0 | 1.4 | 0.4 |
| Switzerland | 2.6 | 0.8 | 1.3 | 3.2 | 1.7 | 0.2 | 1.0 | 0.6 |
| United Kingdom | 7.0 | 3.4 | 8.1 | 7.9 | 2.9 | 3.7 | 4.0 | 1.1 |
| United States | 15.5 | 4.6 | 17.0 | 18.0 | 4.1 | 5.6 | 5.4 | 0.7 |
